# Targeting Alzheimer’s Disease Neuro-Metabolic Dysfunction with a Small Molecule Nuclear Receptor Agonist (T3D-959) Reverses Disease Pathologies

**DOI:** 10.4172/2161-0460.1000238

**Published:** 2016-06-03

**Authors:** Ming Tong, Cesar Dominguez, John Didsbury, Suzanne M de la Monte

**Affiliations:** 1 Liver Research Center, Warren Alpert Medical School of Brown University, Providence, RI, USA; 2 Divisions of Gastroenterology, Warren Alpert Medical School of Brown University, Providence, RI, USA; 3 Department of Neuropathology, Warren Alpert Medical School of Brown University, Providence, RI, USA; 4 Department of Medicine, Warren Alpert Medical School of Brown University, Providence, RI, USA; 5 Department of Pathology, Warren Alpert Medical School of Brown University, Providence, RI, USA; 6 Department of Neurology, Warren Alpert Medical School of Brown University, Providence, RI, USA; 7 Department of Neurosurgery, Rhode Island Hospital and the Warren Alpert Medical School of Brown University, Providence, RI; 8 Williams College, Williams, MA, USA; 9 T3D Pharmaceuticals, Raleigh Durham, NC, USA5Australian School of Advanced Medicine, Macquarie University, Sydney, Australia

**Keywords:** Alzheimer, T3D-959, PPAR agonist, Type 3 diabetes, Streptozotocin, Motor function, Cerebellum, Neurodegeneration

## Abstract

**Background:**

Alzheimer’s disease (AD) could be regarded as a brain form of diabetes since insulin resistance and deficiency develop early and progress with severity of neurodegeneration. Preserving insulin’s actions in the brain restores function and reduces neurodegeneration. T3D-959 is a dual nuclear receptor agonist currently in a Phase 2a trial in mild-to-moderate AD patients (ClinicalTrials.gov identifier NCT02560753). Herein, we show that T3D-959 improves motor function and reverses neurodegeneration in a sporadic model of AD.

**Methods:**

Long Evans rats were administered intracerebral (i.c.) streptozotocin (STZ) or normal saline (control) and dosed orally with T3D-959 (1.0 mg/kg/day) or saline for 21 or 28 days. Rotarod tests evaluated motor function. Histopathology with image analysis was used to assess neurodegeneration.

**Results:**

T3D-959 significantly improved motor performance, and preserved both cortical and normalized white matter structure in i.c STZ-treated rats. T3D-959 treatments were effective when dosed therapeutically, whether initiated 1 day or 7 days after i.c. STZ.

**Conclusion:**

T3D-959’s targeting neuro-metabolic dysfunctions via agonism of PPAR delta and PPAR gamma nuclear receptors provides potential disease modification in AD.

## Introduction

Effective disease modifying therapy is critically needed for the treatment of Alzheimer’s disease (AD). Despite extensive research and large-scale, long-term treatment with drugs that target symptoms including acetylcholinesterase inhibitors, NMDA receptor antagonists and amyloid vaccines, the clinical course of AD has not been significantly remediated. The most likely explanation for these failures is that AD multifaceted and is caused by inter-related cellular, biochemical and molecular abnormalities that culminate in loss of neurons, deposition of amyloid beta, accumulation of phospho-tau-containing neuronal cytoskeletal lesions, activation of cell death cascades, deficits in energy metabolism, mitochondrial dysfunction, increased inflammation, DNA damage and oxidative stress. Importantly, these pathophysiological responses closely resemble effects of insulin resistance and insulin deficiency as occur in diabetes mellitus, except the alterations selectively or predominantly affect the brain. This concept regarding the pathogenesis of AD as a consequence of neuro-metabolic dysfunction led to the term ‘Type-3 Diabetes’ [1,2]. Correspondingly, the earliest abnormalities, preceding the onset of cognitive impairment, include impairments in glucose utilization and energy metabolism [3–5].

After the brain is totally dependent on glucose as an energy source, and reduced brain glucose metabolism is both a hallmark of AD and the future best predictor of cognitive decline [6–9]. Insulin is needed for efficient utilization of glucose by brain cells, and is the master hormone whose signaling regulates major biological responses including: Cell growth, neuronal and oligodendroglial survival, neuronal plasticity, energy metabolism, acetylcholine production, inhibition of oxidative stress, and myelin maintenance. Reductions in brain insulin signaling due to insulin deficiency or insulin receptor resistance could account for the majority of molecular, biochemical, and histopathological lesions, as well as cognitive impairment in AD [2,10–18]. Furthermore, disruption of neuronal insulin signaling networks enhances apoptosis [19,20], promotes oxidative injury induced by Aβ_1–42_ [21], increases secretion of Aβ_1–42_ [22], blocks removal of extracellular Aβ-oligomers [23] and increases plaque burden [24].

Growing evidence suggests that brain insulin resistance promotes or triggers key pathologies in AD [2,3,11,25–34], corresponding with the observed changes in levels of insulin signaling molecules in AD forebrains and associated declines in memory [2,11,12,27,32,34–36]. What is particularly alarming is that peripheral insulin resistance, as occurs in Type 2 diabetes mellitus (T2DM), contributes to AD and cognitive impairment by reducing brain insulin uptake and increasing brain levels of Aβ, tau-phosphorylation, oxidative stress, pro-inflammatory cytokines, advanced glycation end products, dyslipidemia, and apoptosis [12,13,37–41]. However, AD shares many pathophysiological features including insulin resistance, oxidative stress, inflammation amyloid aggregation and cognitive impairment with T2DM and other peripheral insulin resistance diseases such as metabolic syndrome and non-alcoholic fatty liver disease [25,26,37,42,43], yet these disease processes can occur independently or overlap with one another. One consideration is that insulin resistance diseases share the same or similar underlying causes, but individual factors govern the targeting of specific organ-systems.

Given the spectrum of abnormalities in AD, disease remediation will likely require treatment of multiple molecular and biochemical targets [44]. Peroxisome proliferator-activated receptors (PPARs) are nuclear hormone receptors that function as transcription factors and regulate gene expression [45–48]. PPAR-β/δ, the primary target of T3D-959, are highly expressed in brain, and PPAR-γ, the secondary target of T3D-959, is widely expressed throughout the body. Hetero-dimerization of PPARs with retinoid x receptors regulates target genes [45–48], and the attendant alterations in intracellular signaling enhance energy metabolism, cell growth and differentiation, and inhibit inflammation and oxidative stress [49–53]. Since previous studies showed that PPAR-β/δ agonists can effectively treat experimental STZ [10,54] and 5XFAD [55] models of AD, such agonists are attractive therapeutic targets for AD. Since PPAR-β/δ is abundantly expressed in the brain, and both PPAR-β/δ and PPAR-γ [56,57] agonists exhibit therapeutic efficacy in experimental AD models, dual activation of their receptors may provide enhanced therapeutic benefit in humans with AD.

T3D-959 is a brain penetrating dual nuclear receptor agonist in which PPAR-δ is the primary target (human ED_50_=19 nM) while PPAR-γ is the secondary target (human ED_50_=297 nM). In a previous study, we demonstrated efficacy of T3D-959 for preserving spatial learning and memory and preventing cellular and molecular indices of neurodegeneration in an established experimental model of sporadic AD [54]. The present work characterizes the structural and functional therapeutic effects of T3D-959 on i.c. STZ-induced cerebellar degeneration in the same model.

The cerebellum was studied because it is targeted by neurodegeneration in AD [2,58], insulin signaling is an important regulator of cerebellar structure and function [59], and the cerebellum is a major target of brain insulin resistance, irrespective of cause [17,18,30,44,59–62]. Since 1999, at least 53 human research articles have been published demonstrating that besides its role in coordination of voluntary movement, gait, posture, speech and motor function, the cerebellum helps control cognitive functions that require precise timing and behavior [63–67]. It is also believed that cell loss in the cerebellum could be related to coordination deficits that often occur in mid-to-late stages of AD [67–71]. The present work was designed to increase understanding of the nature and degree to which a novel δ/γ PPAR agonist might be beneficial for treating or reducing severity of neurodegeneration in structural targets of brain insulin resistance in AD.

## Methods

### Intracerebral Streptozotocin (STZ)

Intracerebral (i.c.) treatment with STZ was used to cause brain metabolic dysfunction and insulin resistance as occur in human sporadic AD [3,16]. Long Evans male and female rats (8–12/group) were anesthetized by intraperitoneal (i.p.) injection of ketamine (100 mg/kg)/xylazine (10 mg/kg) and administered i.c. STZ (0.9 mg/kg) or saline (1–2 μL volumes) into the lateral ventricle (1 mm caudal, 2 mm lateral to the bregma) as previously described [72]. Rats were administered 5 mL/kg of subcutaneous sterile saline to avoid post-operative dehydration, and kept warm using a temperature-controlled blanket until they were fully recovered.

### Formulation and administration

T3D-959 was formulated as a solution in 0.9% NaCl at a concentration of 4.44 mg/ml and prepared fresh daily. Rats were treated by oral gavage with 1.0 mg/kg T3D-959 or normal saline vehicle, once daily from 1 day or 7 days after the i.c. STZ or vehicle treatments. Oral gavage was performed using a ball ended feeding needle to inject drug or vehicle.

### Rotarod studies

We used rotarod tests to assess long-term effects of i.c. STZ treatment and the therapeutic effects of T3D-959 on motor function [73]. Rats were administered 10 trials at incremental speeds up to 10 rpm, with 10 min rest between trials. The latency to fall was automatically detected and recorded with photocells placed over the rod. Trials were stopped after 30 s to avoid exercise fatigue. Data from trials 1–3 (2–5 rpm), 4–7 (5–7 rpm), and 8–10 (8–10 rpm) were culled and analyzed using the Mann-Whitney test.

### Histological and image analysis studies

**Figure 1:**
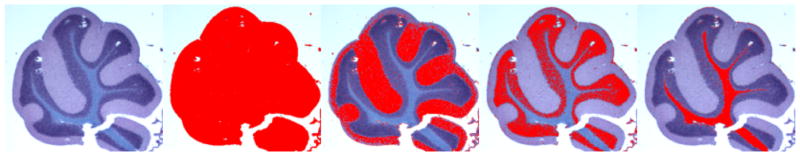
Image analysis approach. (A) Histological sections of the cerebellar vermis were stained with LHE to highlight the cerebellar cortex and white matter. Image J/Fiji software was used to measure (highlighted in red) (B) Total cross-sectional areas of the vermis, (C) The molecular layer, (D) Granule cell layer, and (E) White matter.

The rats were sacrificed by isoflurane inhalation. Cerebella were cut in the mid-sagittal plane, weighed, immersion fixed in 10% formalin, and embedded in paraffin. Histological sections (4 μm thick) generated 5 mm from the midline were stained with Luxol Fast Blue, hematoxylin and eosin (LHE) and used for histopathological assessments and image analysis. Assessments of regional brain atrophy can be achieved by several approaches that may differ in sensitivity, including measurement of cortical thickness and cross-sectional area [74]. Therefore, two approaches were used to extract information about cortical and white matter thicknesses in relation to i.c. STZ and T3D-959 treatment: 1) image analysis to measure cross-sectional areas; and 2) semi-quantitative assessments of cortical and white matter thicknesses and cellularity within the granule and Purkinje cell layers. ImageJ/Fiji was used to determine cross-sectional areas of the entire slice, molecular and granule cell layers, and white matter (Figure 1). In addition, histological sections were scored with respect to uniformity of thickness or cellularity of the molecular, granule cell, Purkinje cell, and white matter regions of the vermis using a semi-quantitative grading system (Table 1). All slides analyses were performed under code.

### Statistics

Graphs depict group means and standard deviations. Inter-group comparisons were made using one-way or two-way analysis of variance (ANOVA) and the Tukey post hoc test (GraphPad Prism 6, San Diego, CA).

## Results

### T3D-959 treatments restore i.c. STZ-mediated impairments of motor function

**Figure 2:**
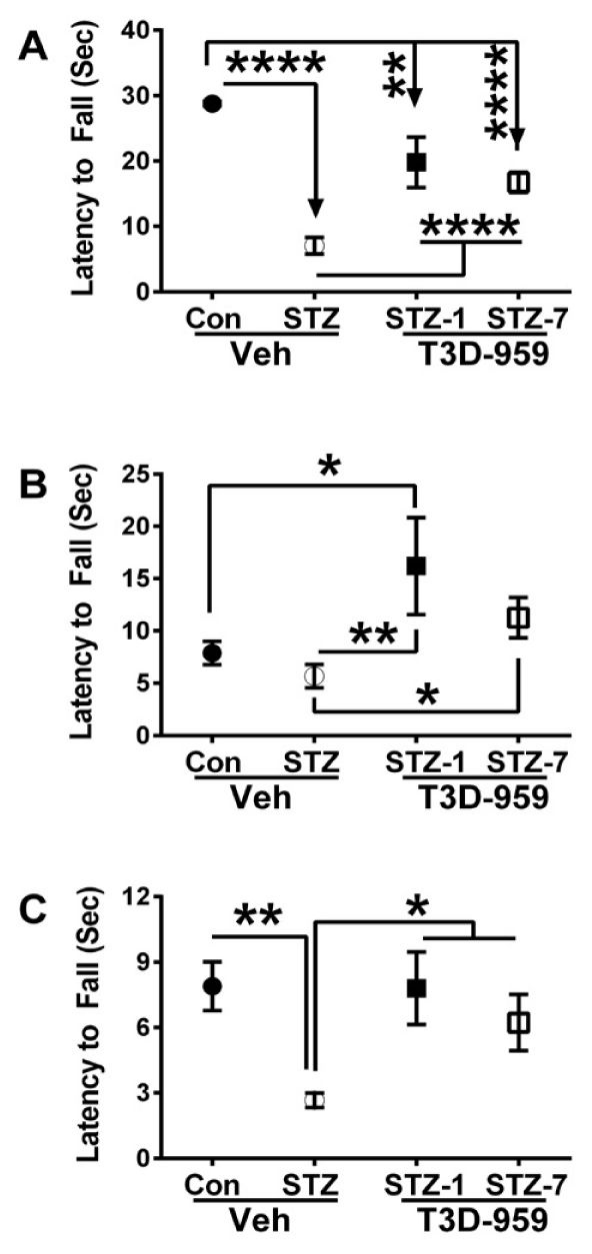
T3D-959 rescue of i.c. STZ-mediated of motor impairment. Long Evans rats were administered i.c. STZ or normal saline (Control). The control rats and a subset of the i.c. STZ rats were gavage with saline. Two other sub-groups of i.c. STZ rats were gavage with a single daily dose of 1.0 mg/kg of T3D-959 beginning 1 day or 7 days after the i.c. STZ injections. Sixteen days later, the rats were subjected to Rotarod testing with 10 incremental speed trials. Latency to fall was measured optically. Data culled from Trials (A) 1–4, (B) 5–6, and (C) 8–10 are depicted in the graphs (mean ± S.E.M. per group/trial). Inter-group comparisons were made using the Mann-Whitney test. *P<0.05; **P<0.01; ****P<0.0001.

Rotarod data were grouped and analyzed using the Mann-Whitney test. With increasing difficulty of the trials (based on rotation speed of the rod), latency to fall declined in all groups (Figure 2). However, with regard to the least challenging series of trials (#1–#4), the mean latency to fall was significantly reduced by i.c. STZ relative to control (Figure 2A). Treatment with T3D-959 improved performance in the STZ group, but the difference relative to control was still significant. For intermediate-level trials (#5–#7), T3D-959 treatment significantly increased mean latency to fall in both control and STZ-exposed rats relative to corresponding vehicle-treated rats (Figure 2B). For the most challenging group of trials (#8–#10), latency to fall was significantly shorter in the STZ-exposed relative to the other 3 groups, and performances of vehicle-treated controls, T3D-959 treated controls, and T3D-959 treated STZ-exposed rats were similar to each other (Figure 2C).

### T3D-959 effects on STZ-associated cerebellar degeneration

**Figure 3:**
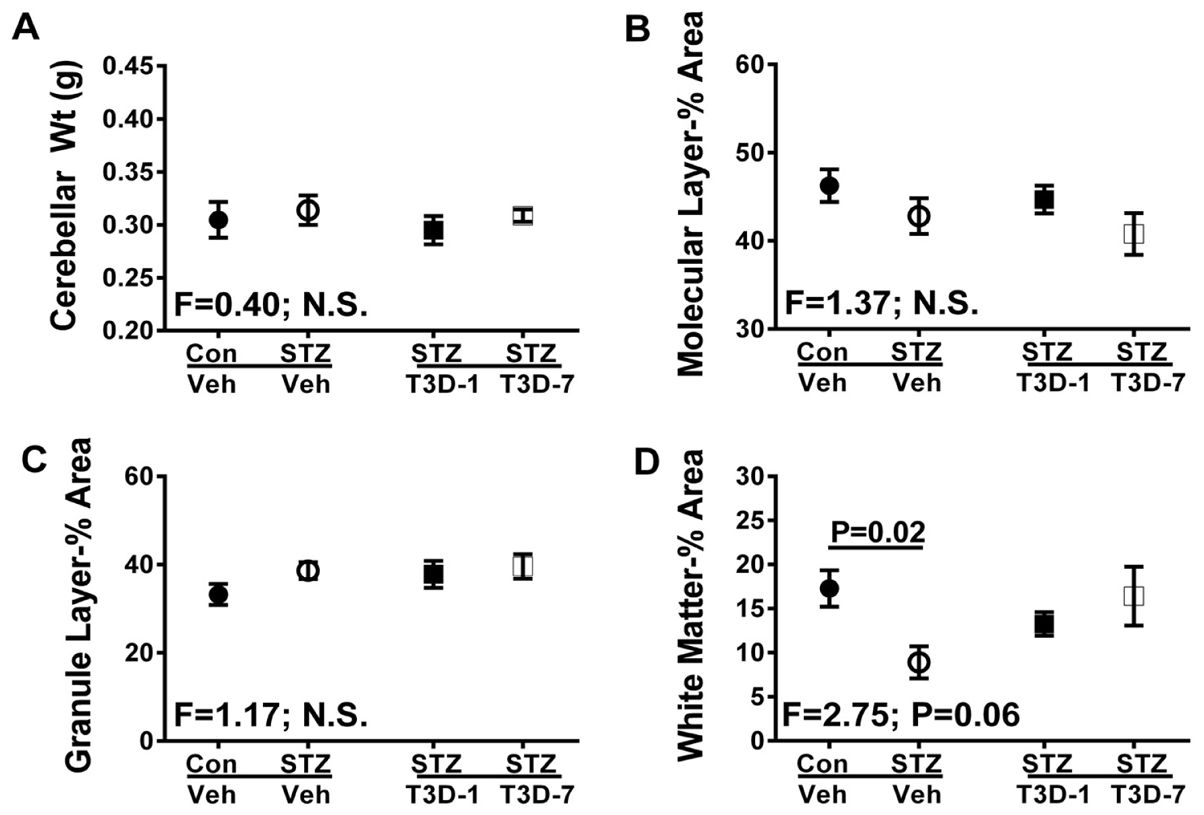
Effects of T3D-959 on i.c. STZ-induced alterations in cerebellar structure. Long Evans male rats (4-weeks old) were used to generate a sporadic model of AD by i.c. STZ. The rats (n=6–8 per group) were treated with saline or 1 mg/kg of T3D-959 by daily oral gavage. Rats were sacrificed on Experimental day 28. (A) Fresh cerebellar weights in rats treated with i.c. saline (Con), ic-STZ (STZ), T3D-959 beginning 1 day after i.c. STZ (STZ T3D-1), or T3D-959 beginning 7 days after i.c.-STZ (STZ T3D-7). After formalin-fixation, cerebella were cut in the mid-sagittal plane and embedded in paraffin. Histological sections (4 μm thick) stained with hematoxylin and eosin (H&E) were used for image analysis (Image-J) to measure relative cross-sectional areas (percentages of overall area) of the (B) molecular layer, (C) granule cell layer, and (D) white matter. All graphs depict the mean ± S.E.M. of results. Inter-group comparisons were made by one-way ANOVA with the Tukey post-hoc significance test. F-ratios are indicated in the panels. Significant inter-group differences are specified.

There were no statistically significant inter-group differences in the mean cerebellar weights (Figure 3A), or the mean relative cross-sectional areas of the molecular (Figure 3B) or granule (Figure 3C) cell layers. In contrast, i.c. STZ significantly reduced the mean relative cross-sectional area of white matter. Treatment with T3D-959 from 1 or 7 days after i.c. STZ prevented white matter atrophy; however, the 7 day delayed treatment results more closely approximated the findings in controls (Figure 3D).

### T3D-959 effects on STZ-mediated cerebellar histopathology

**Figure 4:**
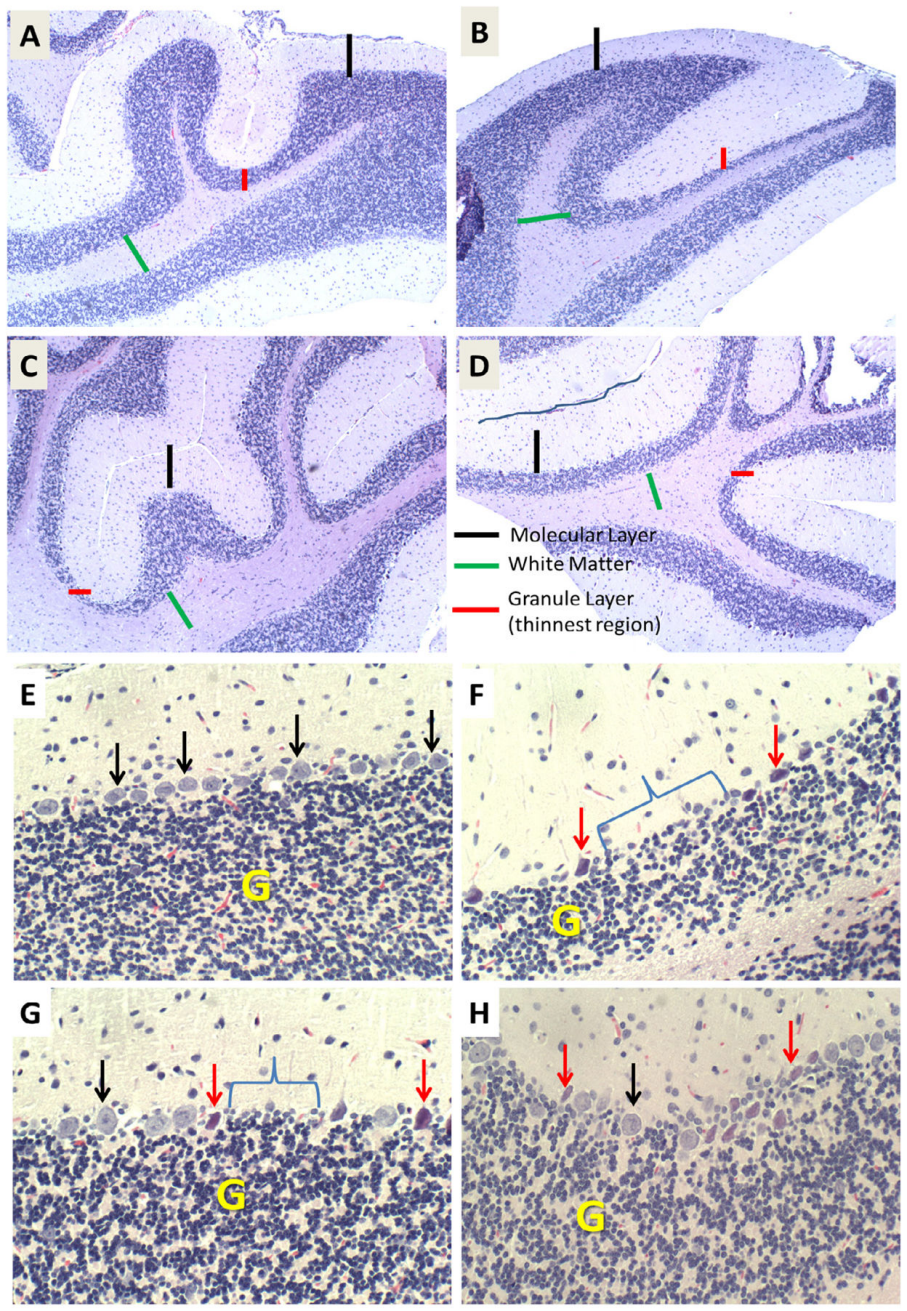
T3D-959 Prevents STZ-induced neurodegeneration. H&E stained sections of the cerebellar vermis from rats treated with (A, E) i.c. saline or (B–D, F–H) i.c. STZ, and then treated with (A, B, E, F) vehicle (saline) or (C, D, G, H) 1 mg/kg/day T3D by gavage. T3D after delays of (C, G) 1 day or (D, H) 7 days. Rats were sacrificed 4 weeks after i.c. STZ or vehicle injections. Cerebella were fixed in formalin, embedded in paraffin and sectioned (4 μthick). (A, E) Control cerebella had relatively thick and uniform molecular (black bars) and granule cell (red bars, yellow ‘G’), (E) well populated Purkinje cell layers (black arrows), and uniform white matter cores (green bars). Relative to control, i.c. STZ treatment caused cortical atrophy with (B) thinning of the molecular and (B, F) granule cell layers, (F) loss (bracket) and on-going necrosis (red arrows) of Purkinje cells, and modest reductions in white matter thickness. (C, D, G, H) T3D-959 normalized or expanded the molecular layer and increased white matter core thickness, reduced cell loss and necrosis in the Purkinje layer, but did not reverse the irregular thinning and neuronal loss in the granule cell layer. Original magnification: (A–D) 100x; (E–H) 425x.

**Figure 5:**
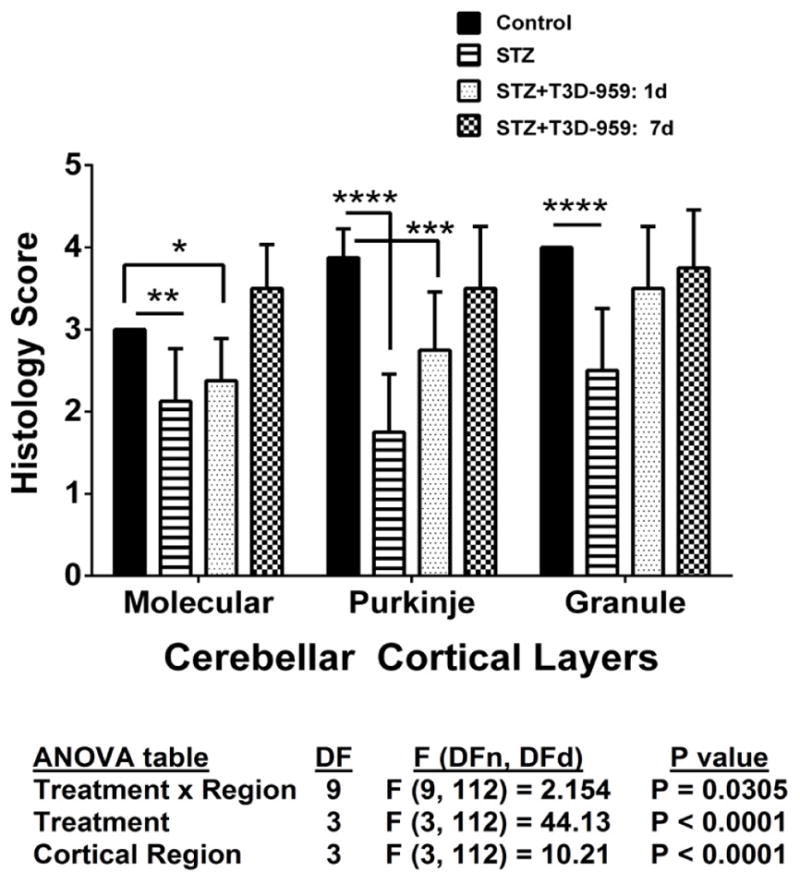
Semi-quantitative grading of cerebellar histopathology. H&E stained sections of cerebellar vermis were scored under code using semi-quantitative assessments of the uniformity and thickness of the molecular layer, cellularity within the Purkinje layer, and thickness and cellularity of the granule cell layer (Table 1). The mean ± S.D. scores corresponding were compared across experimental groups by two-way ANOVA (results tabulated below graph) with the Tukey post-hoc multiple comparisons test (*P<0.05; **P<0.01; ***P<0.001; ****P<0.0001).

Although the image analysis of cross-sectional areas demonstrated white matter atrophy in the i.c. STZ group and therapeutic responses to T3D-959, visual inspection of the histopathology revealed clear inter-group differences in cortical layer cellularity and thickness that were not detected by that approach. Therefore, systematic analysis (Figure 4) and scoring (Figure 5) of cellular pathology in the cerebellar vermis under code was used to provide more detailed assessments of i.c. STZ’s effects and the therapeutic responses to T3D-959. In all samples, the cerebellar architecture had the expected organization including an outer molecular layer, middle Purkinje cell layer, and thick, densely populated granule cell layer with clearly delineated white matter cores. In controls, the cerebellar architecture was relatively uniform within each layer and exhibited minimal or no evidence of cell injury or loss (Figures 4A and 4E). The i.c.-STZ treatment caused cortical atrophy with thinning of the molecular layer, reduced populations of Purkinje cells with large gaps corresponding to regions of neuronal loss, as well as multiple neurons exhibiting shrinkage and eosinophilia reflecting ongoing necrosis. In addition, i.c. STZ caused irregular thinning of the granule cell layer and white matter cores (Figures 4B and 4F). T3D-959 treatments initiated either 1 day or 7 days post i.c. STZ increased the molecular layer thickness, reduced neuronal loss and necrosis in the Purkinje layer, and expanded the white matter core thickness (Figures 4C, 4D, 4G and 4H). But, did not restore the thickness of the granule cell layer. Although T3D treatment conspicuously increased cell density within the granule layer, the granule cell layer thickness remained irregularly thinned. These results suggest that T3D may be neuroprotective for both granule and Purkinje cells.

### Semi-quantitative histological grading of cerebellar structure

Since the histological abnormalities in the cortex were not well reflected by the image analysis results, we evaluated the tissue sections using a semi-quantitative grading scheme to systematically assess effects of STZ and T3D treatments on cerebellar vermis architecture (Table 1). H&E stained sections of cerebellar vermis were scored under code using semi-quantitative assessments (Table 1). The mean ± S.D. scores corresponding to uniformity and thickness of the molecular layer, cellularity of the Purkinje layer, and thickness and cellularity of the granule cell layer are depicted in Figure 5. Two-way ANOVA demonstrated significant inter-group differences with respect to all three regions of cortex. STZ treatment significantly reduced the thickness and uniformity of the molecular layer, and cellularity in the Purkinje and granule cell layers relative to control (Figure 5). The differences in molecular layer thickness and Purkinje cellularity relative to control remained significant for the group that was treated withT3D-959 beginning 1 day after i.c. STZ. However, cellularity and thickness uniformity of the granule cell layer were restored by early treatment with T3D-959. Furthermore, T3D-959 treatment beginning 7 days after i.c. STZ restored or preserved the integrity of all three cortical layers relative to control.

## Discussion

Since 2005, over 60 published works have shown that the i.c. STZ model of brain diabetes mimics most aspects of sporadic AD with respect to molecular, biochemical, histopathological, and/or neurobehavioral abnormalities [1,54,75–78]. In this regard, the i.c. STZ model causes amyloid-β deposition, pTau accumulation, cortical-limbic pathway degeneration, deficits in spatial learning and memory, neuro-inflammation, and oxidative stress in the brain, including in regions that are characteristically damaged in AD. In addition, the data show the importance of brain insulin resistance as a mediator of neurodegeneration and amyloid deposition, and vice versa [28,29,36,44,79,80].

AD like diabetes mellitus is associated with insulin resistance, except brain rather than skeletal muscle is the principal organ. In the brain, insulin is a key regulator of glucose utilization and signal transduction networks that mediate cell growth, plasticity, metabolism, neuronal survival, myelin maintenance and acetylcholine biosynthesis, and it inhibits oxidative stress and apoptosis [1,14,15,30,44,59]. Proof of principle for this concept has been provided by experiments in which i.c. administration of STZ, a pro-diabetes toxin, was shown to cause AD-type neurodegeneration [16], and early treatment with PPAR agonists was demonstrated to prevent cognitive impairment and neurodegeneration [10]. The PPAR-δ agonist proved to be considerably more neuroprotective in preserving cognitive function and hippocampal/temporal lobe structure compared with the PPAR-γ agonist [10], corresponding with the greater abundance of δ versus γ receptor expression in brain [55].

A major advantage of PPAR agonists is that they mediate their effects within the nucleus, circumventing impairments in insulin signaling caused by reductions in surface receptor binding and receptor tyrosine kinase activation, which are features of AD [2,11]. The neuroprotective effects of PPAR-δ and PPAR-γ agonists overlap but are non-identical with respect to downstream insulin-responsive targets [45–48]. T3D-959 is small molecule hybrid PPAR-δ/γ agonist that can be administered as a once daily oral dose and exhibits a high degree of CNS penetrance.

PPAR agonists of all classes that bind to nuclear hormone receptors can cause cellular harm at high concentrations a purported selective. In the manuscript by Peri et al. [81] and another by Rohn et al. [82], the concentrations of PPAR-gamma agonist at high caused neuronal apoptosis at 10 micro-M but not 500 nM. Our experimental conditions produced nM tissue concentrations of T3D-959 [54]. Interestingly, the manuscript by Rohn et al. [82] does show nice neurite process extension and improved neuronal viability at the lowest doses of the PPARgamma agonist used, 15-PGJ2. Furthermore, 15d-PGJ2 [82] is not a selective PPAR gamma agonist as it also interacts with GPR44; acting through this receptor inhibits hair growth. The high doses of 15d-PGJ2 needed to induce apoptosis are not biologically relevant and can produce off-target effects. In addition, this compound acts as a PGD2 receptor agonist with an EC_50_ of 10nM, and can have PPAR gamma–independent effects [83], calling into question the conclusion that the PPAR gamma activation caused neuronal apoptosis. In contrast, T3D-959 has been demonstrated to increase cell survival (see Figure 4a and article by Tong et al. [54]).

The preclinical studies reported herein assessed the neurobehavioral effects of T3D-959 in an established model of sporadic AD in which adult Long Evans rats were treated by i.c. STZ which is known to cause neurodegeneration, impairments in brain insulin and insulin-like growth factor (IGF) signaling, and increased oxidative stress [10,16]. In addition to assessing T3D-959’s efficacy, we examined responses to early and late therapeutic intervention. The delayed time point of initiating treatment addresses the fact that many people are first diagnosed at clinically intermediate rather than early stages of disease. At the same time, it was important to assess responses to early intervention because as diagnostic tools improve, early treatment protocols will become feasible.

The rotarod tests demonstrated that i.c. STZ impaired cerebellar motor function and that T3D-959 prevented this effect along with neurodegeneration. It was of further interest that these therapeutic responses to T3D-959 were similar and slightly better when treatment was initiated 7 days as opposed to 1 day post i.c. STZ. Histopathological studies demonstrated that the i.c. STZ mediated neuronal loss in Purkinje and granule cell layers and atrophy of white matter were reversed by T3D-959 treatment. Therefore, the neuroprotective effects of T3D-959 correlate with preservation of motor function.

The therapeutic effects of T3D-959 on cerebellar structure and function correspond with the abundant expression of insulin receptors and prominence of insulin signaling networks in the cerebellum [59]. Previous studies using various experimental models showed that inhibition of insulin signaling in the cerebellum or cerebellar neurons correlates with neuronal loss and impaired motor function [17,60,62]. In addition, earlier studies showed that treatment with a PPAR-δ agonist effectively restored cerebellar structure and function [10], consistent with findings in the present study. An additional novel finding was the reversal of white matter atrophy in T3D-959 treated i.c. STZ exposed rats. This observation is of particular interest because myelin-producing oligodendrocytes are maintained by insulin/IGF signaling, and white matter atrophy is an early feature of AD [84–86]. Altogether, these data suggest that motor impairments mediated by neuronal loss and white matter atrophy in AD may be ameliorated by T3D-959 therapy.

In conclusion, this study demonstrates that T3D-959 has cleared therapeutic and neuroprotective effects in an established model of sporadic AD. The main effects were associated with improved (normalized) motor function and reversal of cerebellar degeneration. Importantly, therapeutic effects occurred even after a delay in treatment, suggesting that individuals with mild or moderate AD would benefit from this highly effective small molecule drug that has the benefit of oral, once-daily delivery.

## Figures and Tables

**Table 1:** Semi-quantitative grading of cerebellar vermis histopathology.

Cortical Layer	Grade 4	Grade 3	Grade 2	Grade 1
Molecular	Uniformly thick and cellular	Variable thinning, normal cellularity	Variable thinning and reduced cellularity	Uniformly thin
Purkinje	Well populated with histologically intact pyramidal neurons	Isolated neuronal loss or eosinophilic degeneration (necrosis)	Moderate gaps and scattered loss of neurons	Large gaps and conspicuously increased neuronal necrosis
Granule	Uniformly thick and densely cellular	Irregular thinning but densely cellular	Irregular thinning with modest reductions in cell density	Irregular thinning and conspicuous reductions in cell density

H&E stained sections of cerebellar vermis were graded using the semi-quantitative criteria indicated in the table. The mean scores obtained for each layer were compared across the 4 experimental groups by two-way ANOVA (see Figure 5).
